# Comparison of Tendon Development Versus Tendon Healing and Regeneration

**DOI:** 10.3389/fcell.2022.821667

**Published:** 2022-01-24

**Authors:** Peiwen He, Dengfeng Ruan, Zizhan Huang, Canlong Wang, Yiwen Xu, Honglu Cai, Hengzhi Liu, Yang Fei, Boon Chin Heng, Weishan Chen, Weiliang Shen

**Affiliations:** ^1^ Department of Orthopedic Surgery, The Second Affiliated Hospital, Zhejiang University School of Medicine, Hangzhou, China; ^2^ Orthopedics Research Institute of Zhejiang University, Hangzhou, China; ^3^ Institute of Sports Medicine, Zhejiang University, Hangzhou, China; ^4^ Key Laboratory of Motor System Disease Research and Precision Therapy of Zhejiang Province, Hangzhou, China; ^5^ Central Laboratory, Peking University School of Stomatology, Bejing, China; ^6^ Dr. Li Dak Sum and Yip Yio Chin Center for Stem Cell and Regenerative Medicine, Zhejiang University, Hangzhou, China; ^7^ China Orthopaedic Regenerative Medicine (CORMed), Hangzhou, China

**Keywords:** tendon, development, healing, regeneration, regulation, comparison

## Abstract

Tendon is a vital connective tissue in human skeletal muscle system, and tendon injury is very common and intractable in clinic. Tendon development and repair are two closely related but still not fully understood processes. Tendon development involves multiple germ layer, as well as the regulation of diversity transcription factors (Scx et al.), proteins (Tnmd et al.) and signaling pathways (TGFβ et al.). The nature process of tendon repair is roughly divided in three stages, which are dominated by various cells and cell factors. This review will describe the whole process of tendon development and compare it with the process of tendon repair, focusing on the understanding and recent advances in the regulation of tendon development and repair. The study and comparison of tendon development and repair process can thus provide references and guidelines for treatment of tendon injuries.

## 1 Introduction

Tendons are the dense connective tissue that connects muscle to bone and primarily serve to transmit muscle contraction and anchor muscle (Benjamin and Ralphs, 1997). Mature tendons consist of tightly arranged collagen fibers of different diameters. Tenocytes distribute among the fibrils and synthesize a large count of extracellular matrix (ECM) that is composed mainly of collagens and proteoglycans, which serves to lubricate and assemble collagen fibers (Asahara et al., 2017).

Current research on the development of musculoskeletal system is relatively comprehensive, except connective tissue like ligaments and tendons. The master genes regulating the bone, skeletal muscle, and cartilage lineages have been proved as the Runt-related transcription factor *Runx2* and the transcription factor *Dlx5* (bone) (Komori et al., 1997; Otto et al., 1997), the bHLH transcription factors *MyoD*, *Myf5*, and *Mrf4* (muscle) (Weintraub, 1993; Delfini and Duprez, 2004; Kassar-Duchossoy et al., 2004), the SRY-box transcription factor *Sox9* (cartilage) (Akiyama et al., 2002; Takimoto et al., 2012). However, identification of master genes regulating tendon lineage is still ongoing. Although it has been demonstrated that *SCX*, *TNMD*, *MKX* and other transcription factors or relative specific proteins profoundly influence tendon development, their tissue specificity in tendon has not been fully demonstrated. For example, Scx is also known to take effect in the cells of heart valves, lungs and other ECM-rich tissues (Levay et al., 2008; Ramírez-Aragón et al., 2020). Many studies point to the hypothesis that tenocytes and chondrocytes have very similar origins (Soeda et al., 2010; Lee et al., 2020). This is understandable, because tendons and cartilage are both relatively high ductility and low rigidity tissues. Thus, it may be the balance between different factors what makes the different in progenitors and it is of great significance to explore the signals that regulate these factors.

The incidence of tendon injuries and tendinopathy has been increasing substantially in recent decades. Tendon or ligament injuries constitute almost half part of sports and physical activity-related injuries, with 30–50 million new incidents occurring annually worldwide (Ljungqvist et al., 2008). Hence, the healing of tendon injury has gradually become a research hotspot. To date, the majority of studies for repair of tendon injury are focused on scar-associated healing models, since adult tendon does not normally regenerate (Katzel et al., 2011; Dyment et al., 2014; Ackerman et al., 2019b). Experimental results showed that adult tendon healing was characterized by continuous infiltration of aSMA+ cells, loss of tendon cell proliferation or recruitment, abnormal differentiation of chondrocytes, and loss of tendon functional (Kaji et al., 2020). There are research studies showing that the Young’s moduli in healed rabbit Achilles tendons is nearly 80% of its uninjured value (Nagasawa et al., 2008), while the stiffness and load of healed mouse patellar tendons can reach to only 48 and 63% of the original values respectively (Dyment et al., 2012). While a few research groups have successfully achieved tendon regeneration in model systems such as MRL/MpJ mice, gene editing has a lot challenge and risk in these systems, so it is still a long way from being feasible in humans (Paredes et al., 2018; Paredes et al., 2021). However, embryonic and neonatal tendons have full regenerative capacity, which are driven by tenocyte proliferation, recruitment, and differentiation, leading to complete functional restoration (Howell et al., 2017). Such pattern of tendon regeneration has more in common with the process of tendon development, and many existing studies (Liu et al., 2014; Zhang et al., 2018) have proven that tendon-related transcription factors and signaling pathways, such as Scx and TGFβ pathways, play key roles in the process of tendon repair and regeneration, the mechanism of which needs further study. Therefore, a better understanding of tenogenesis can clarify the mechanisms of tendon disease, injury and healing, regeneration.

This review focuses on the process of tendon development, including the differentiation process from mesenchymal stem cells in gastrula stage to the formation of tendon progenitors and the final differentiation of mature tendon cells which dominate assembly of complete tendons, as well as the expression and regulation of known important tendon genes, and the role of the signaling pathways involved. Given that although the regulatory network of repair and regeneration has some similarities with development, there are also many differences. We will also cover the process of tendon repair and healing, including the recognized process of adult tendon repair and the process of regeneration of embryonic or neonatal tendon, as well as discuss the similarities and differences between tendon development and repair, in order to develop strategies for optimizing tendon repair. The latest studies on the role of transcription factors in the process of tendon development and repair are collated and summarized here, so as to facilitate a better understanding of the latest research progress and to inspire further investigations.

## 2 Formation and Development of Tendons

**FIGURE 1:**
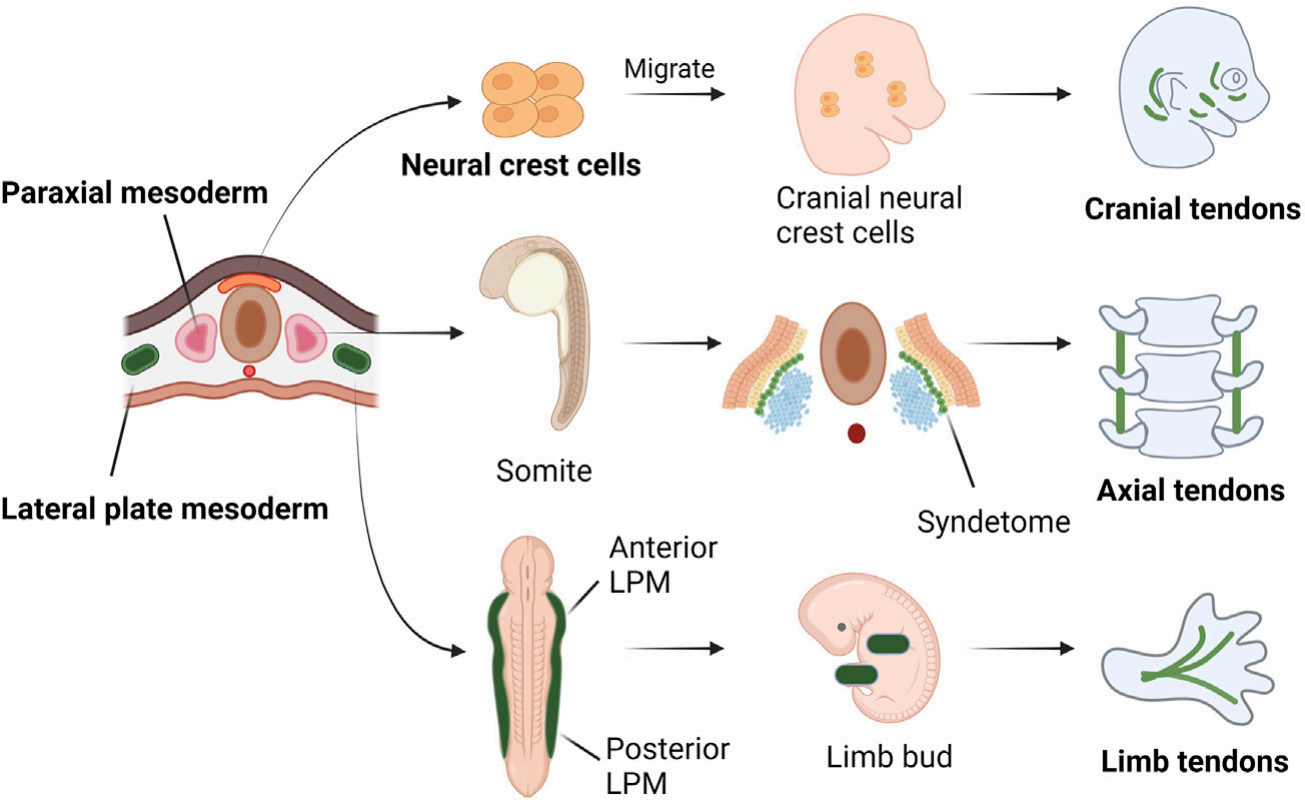
Tendons at different locations have different embryonic origins. Tendons at the cranial region are originated from the neural crest. Some of the nerve crest cells from the ectoderm migrate to the cranial area and differentiate into tendon progenitors. The axial tendons are derived in the syndetome, a dorsolateral stripe of the sclerotome at the junction between adjacent myotomes. At a cross section of embryo, the LPM is formed on either side of the central axis and is subdivided into anterior and posterior parts, corresponding to four limb buds. In limb buds, tendon progenitors are derived from the mesenchyme directly under the ectoderm, in locations that follow the proximal-to-distal outgrowth of the limb bud.

During embryonic development of vertebrates, the embryo goes through a phase of development called gastrulation. During this phase, the embryo differentiates into three germ layers: ectoderm, mesoderm and endoderm. Tendon is mainly originated from the neural crest cells of the ectoderm, the paraxial mesoderm and the lateral plate mesoderm. Tendons at the craniofacial region are derived from the neural crest (Chen and Galloway, 2014). The axial tendons are derived from the syndetome, one of the four compartments of somites (Schweitzer et al., 2001; Brent et al., 2003). In the limb bud, tendon progenitor cells are induced directly from the mesenchyme below the ectoderm and grow along the limb bud proximal to distal (Schweitzer et al., 2001) (Figure 1).

### 2.1 Classification of Tendons

Based on function, tendon can be classified into three distinct types: force-transmitting tendons, intermuscular tendons, and anchor tendons. The most visible force-transmitting tendons are the long chord-like tendons of the limbs and tail, which transmits muscle power to move the bone. To perform their biomechanical functions, the extracellular tissue of these force-transmitting tendons is very complex and organized in the form of tightly parallel collagen fibers (Benjamin et al., 2000). Intermuscular tendons are tendinous tissues interconnecting two muscle segments, including the rectus abdominis tendon and the diaphragm tendon (Murchison et al., 2007). Anchor tendons are the starting tendons near the extremities. Their main function is to anchor the muscle to the starting point of the bone. An example of an anchoring tendon is the tendon that connects the intercostal muscle to the ribs (Murchison et al., 2007).

Based on the location of the attached muscle, tendons can also be classified into three types: craniofacial tendons, axial tendons and limb tendons. Due to different functions, the force-transmitting tendons are mainly distributed in the limbs, craniofacial tendons as well as tail tendons, while the anchoring tendons are distributed in all three locations, and the intermuscular tendons are distributed within the trunk. These three types of tendons tissues are derived from different origins, with the relevant information described above.

### 2.2 The Development of Tendons in Animal Models

#### 2.2.1 Limb Tendons

##### 2.2.1.1 The Origin of Limb Tendons: The Lateral Plate Mesoderm

The tendons in limbs are originated in the lateral plate mesoderm (LPM). LPM condenses into bilateral cell sheets at the lateral edge of the immature vertebrate embryo, which is so-called lateral plate. Previously, the LPM was also described as leading-edge, ventral, lateral, ventrolateral or visceral mesoderm (Prummel et al., 2020).

The lateral plate mesoderm is located at the most lateral region of the mesoderm. After the formation of the gastrula, LPM exhibits its characteristic structure: bilateral plates of LPM progenitor cells form laterally in the embryo and are subsequently segmented into specialized cell fate domains along the A-P and medial-lateral axes (McDole et al., 2018; Prummel et al., 2019). During the process of segmentation, the LPM further divided into anterior (ALPM) and posterior (PLPM) regions, while cell regions on both sides gradually differentiate into progeny cell fates and show different patterns of gene expression. Throughout this process, BMP and Nodal signaling pathways play an important role in lateral plate mesoderm formation, while FGF, Wnt and retinoic acid (RA) signaling also affect the formation of LPM (Arnold and Robertson, 2009).

Forelimb and hindlimb buds develop along the A-P axis at different locations. RA signaling pathway and Hox gene are participated in the correct localization of the progenitor field (Moreau et al., 2019). LPM expressing Tbx5 and Tbx4 interacts closely with epidermal FGF ligand secretion, among other factors, promoting limb formation in ALPM and PLPM, respectively (Nishimoto and Logan, 2016). These limb buds consist of an ectodermal membrane that contains the proliferating mesenchyme with differentiation potential and become the source of further differentiation into the origin of limb connective tissue-tendon progenitor cells (Zeller et al., 2009).

##### 2.2.1.2 The Formation of Limb Tendons

After the condensation of tendon progenitor cells, three pairs of tendon primordia are formed: proximal tendon primordia, intermediate primordia and distal primordia. These primordia are subdivided into single tendons related with each joint. In the hind limbs, proximal, intermediate, and distal tendon primordia generate thigh, shank and foot muscles respectively (Kardon, 1998).

Schweitzer et al. (2001) were the first to discover the expression of *Scx* in tendon cell lineages. In chicken embryos, *Scx* transcripts are found in all muscle-to-bone attachment sites in stage 21 embryos, which is the first phase. Notably, Scx expression is present in all limb tendons, both distal and proximal. In the second phase, stage 25–27, the Scx expressing cells merge to form complex and dynamic patterns that differ from the wing and leg buds and from dorsal and ventral mesenchyme. The third phase of Scx expression, starting at stage 28, is the phase of the first tendon fibers formation. At these stages of limbs development, the autopod enlarges in limbs and cartilage condensations of the digits begin to form. With the initial formation of the digit tendon, Scx is expressed in the broad interstitial streaks formed on the dorsal and ventral side of the digit and just below the ectoderm. Finally, at stages 31 and later, Scx is expressed by all limb tendons. In mice embryos, Scx is expressed in early limb buds. By E9.5-E10, expression of Scx has reached a slightly higher level. Meanwhile, this is the stage of tendon progenitor cell pool formation. Till E14.5, the expression of Scx reaches the distal limb and this expression is further achieved to the maximum at E19, as a good marker for tendon pattern refinement and tendon specific insertion into the respective skeleton elements (Schweitzer et al., 2001).

Subsequent research found that Scx^+^ tendon progenitors are composed of two distinguishing populations according to the expression of Sox9 or not: Scx^+^/Sox9^+^ tendon progenitors and Scx^+^/Sox9^−^ tendon progenitors. These can both eventually differentiate into tendon cells (Soeda et al., 2010; Sugimoto et al., 2013). Moreover, the section of tendon near cartilage or bone contains more Sox9^+^ tendon cells. Thus, Scx^+^/Sox9^+^ progenitors is the main origin of tendons in the surrounding of the vertebrae and ribs, whereas tendons in abdomen are generated from the Scx^+^/Sox9^−^ cell lineage (Sugimoto et al., 2013). In fact, this part of the tendon that is differentiated from Sox9^+^ cells is better known as the tendon-bone interface (enthesis). This will be discussed in detail in the latter sections.

As mentioned above, the force-transmitting tendons are mainly distributed in the limbs as well as in the tail, having a unique way of developing and lengthening compared with anchoring tendons. Research studies have shown that in mouse embryos, long tail and long limb tendons develop via producing of a short anchoring tendon anlage and then tendon lengthen. During this process, new progenitor cells are recruited and differentiated to form tendon tissues, where *Scx* is indispensable (Huang et al., 2019).

The importance of Scx in tendon development is obvious, *Scx* deficient mice were the first observed model of Scx gene influence on tendon development. The other functions and roles of *Scx* will be discussed later. The differentiation and specification of tendons are also influenced and induced by various signaling pathways. Up to date, FGF, TGFβ, Wnt, and mTORC1 signals are known to be participated in limb tendon development. Among these signaling pathways, FGF, TGFβ, and Wnt exert effects on the induction and expression of tendon *Scx* gene, which are the upstream signaling pathways that directly regulate the differentiation of mesenchymal stem cells into tendon progenitor cells, while mTORC1 is the downstream signal for the regulation of extracellular matrix of tendon (Liu et al., 2014; Nakamichi and Asahara, 2021).

#### 2.2.2 Axial Tendons

##### 2.2.2.1 The Origin of Axial Tendons: The Paraxial Mesoderm

Initially, the migration and thickening of the posterior mesoderm cells of the embryo form the primitive streak (PS)/blastopore. Along with the concentration of cells forming the primary streak, a depression appears in the middle of the primary strip, which become the primary sulcus. At the front end of the primary node, the central section of the chimney shapes concave, known as the primary pit. Cells entering the blastocoel through the primary pit migrate anteriorly to form the foregut, head mesoderm and notochord, and cells entering the blastocoel through primary sulcus on either side to form most of the endoderm and mesoderm tissue. The paraxial mesoderm (PM) consists of two tissue strips on either side of notochord. The nascent PM constitutes the presomitic mesoderm at the posterior tip of the embryo, which is a transient tissue that can be further subdivided into an immature posterior and a committed anterior region. The segments of this anterior region are supposed to form the somites. Nodal and BMP4 and other signaling factors promote the formation of the PS and activation of the early mesoderm marker brachyury (T) and Wnt3 signaling in the PM (Beddington and Robertson, 1999; Tam and Loebel, 2007; Ramkumar and Anderson, 2011). The posterior PS and lateral tissues can secrete BMP4, while the axial structures of the embryo produce BMP antagonists, along with the opposite gradients of noggin, antagonizing the action of BMP4 (Winnier et al., 1995; Reshef et al., 1998). Thus, a BMP signaling gradient that controls the mediolateral fates of mesoderm is established. The formation of the paraxial mesoderm is quite sensitive to changes in BMP signaling. Progressively higher levels of BMP signaling is required for all mesodermal types, from the notochord to the extraembryonic mesoderm, for specification (Kishigami and Mishina, 2005).

Next comes the formation of somites. This involves a molecular oscillator which is called the segmentation clock. The principle of segmentation clock is to generate a serious of pulses of Notch, Wnt and FGF signaling to control the periodic generation of somites (Hubaud and Pourquié, 2014). After the formation, somites are divided along the dorsoventral axis into dermomyotome in a dorsal epithelial domain and sclerotome in a ventral mesenchymal domain.

Initially, it was proved that the somites generate to the skeletal muscle, axial skeleton of vertebrate and dorsal dermis. In response to signals secreted from the surrounding tissues, the ventral somite region goes through an epithelio-mesenchymal transition, dividing the somite into two compartments: the dermomyotome and the sclerotome, which are dorsal epithelial layer and ventral mesenchyme respectively. At a slightly later phase, a third compartment forms when cells from the dorsomedial and ventrolateral lips (DML, VLL) of the dermomyotome delaminate from the epithelial sheet, migrate underneath, and re-epithelialize to form the myotome. The remaining myotome gives rise to the skeletal muscle, and the epithelial sheet or dermotome to the dorsal dermis (Borycki and Emerson, 2000; Brand-Saberi and Christ, 2000; Monsoro-Burq and Le Douarin, 2000). However, this process lacks development of connective tissue like tendons. In a later study, a previously undiscovered fourth compartment of somitic which accommodate axial tendon progenitors can be detected, and this fourth compartment is named as “syndetome” (Brent et al., 2003). The discovery of syndetome opened a new stage of human research on the development of somitic tendon.

##### 2.2.2.2 The Formation of Axial Tendons

Syndetome’s developmental origin is later than that of the other three compartments. It was discovered that Scx was faintly expressed at about the 16th stage of chick embryos, while other somitic compartments markers were expressed at the 7th to 9th stage, indicating that the tendon progenitors in syndetome are generated later than other somitic lineages. And it is located between adjacent myotomes sagittal, both ventrolateral and dorsomedial to the sclerotome, which was consistent with observations in mouse and chicken embryos. After induction, Scx is seen at the anterior and posterior somitic borders. By stage 22, expression was remarkably enhanced and continued to extend dorsolateral, and by stage 24, a complete dorsolateral to ventral measurement of body length was Scx positive. In stage 26 embryos, the ventral lateral expression region of Scx is consistent with the formation of early ribs and intercostal muscles (Brent et al., 2003).

Further studies have shown that the syndetome emerges from the sclerotome. Some of the cells in the sclerotome become Scx-expressing cells, and from the ventrolateral region of the somite gradually cover to the ventrolateral region, finally forming the fixed area adjacent to myotomes and sclerotomes (Brent et al., 2003). Besides, another study (Ma et al., 2018) on zebrafish proved that the axial tendons of zebrafish are also developed from the sclerotome. Although the fourth compartment is described in chicken and mouse embryos, this is not clearly observed in zebrafish embryos. The sclerotome of zebrafish embryos is found to be different from those in chicken and mouse embryos. In chick and mouse embryos, the sclerotome is defined as an individual region located in the ventromedial somite. By contrast, the zebrafish sclerotome is initially divided into two separate regions: a ventral region in higher vertebrates and a novel dorsal region. We might speculate that this dorsal region is the region that evolve into the syndetome of higher vertebrates.

#### 2.2.3 Cranial Tendons

##### 2.2.3.1 The Origin of Cranial Tendons: Neural Crest Cells

Neural crest cells (NCCs) are generated within the dorsal-most part of the neuroepithelium at the connection with the surface ectoderm, an area known as the “neural plate border” (Selleck and Bronner-Fraser, 1995). And nascent NCCs detached from the developing neural tube, presenting a mesenchymal feature and migrating to distal parts in the developing embryo. In the cranial area, they generate to a various type of cells and tissues.

The NCCs can be divided into four different populations, referred to as cranial, trunk, cardiac, and vagal NCC. Each subpopulation corresponds to a unique part of a particular cell and tissue type. Cranial neural crest cells (CNCCs) can be further subdivided into front, mid, hind subpopulations, which give rise to most cranial tendons (Yoshida et al., 2008; Parada and Chai, 2015). Lineage tracing of NCCs in zebrafish has demonstrated a stable relevance between the time of cell migration and the ultimate fate of individuals cell (Schilling and Kimmel, 1994). NCCs that migrate early mainly form the skeletal and connective tissues of the cranial region, whereas cells migrate late primarily undergo a neural fate (Calloni et al., 2009).

Initially, BMP, FGF, and Wnt signaling have each been identified as crucial signaling regulators of NCCs formation in various animals such as fish, avian, and amphibians (Crane and Trainor, 2006). Next, more signaling pathways including the above were further proven to be important to the specification of cell-type differentiation within the mammalian NCC lineage. For example, FGF signaling is known to play an important role in facilitating the fate of NCCs towards a skeletogenic type. Previous studies showed that FGF2 increased proliferation and promoted skeletal fate of CNNCs *in vivo* as well as *in vitro* in mouse and avian models (Sarkar et al., 2001; Abzhanov et al., 2003; Li et al., 2010). And TGFβ signaling is revealed as a fate switch controller for SoxE family members that is in control of differentiation fates of cranial NCC. Cranial and trunk NCCs regulated by TGFβ1 low-express Sox10 and differentiate into mesenchymal fates in mouse embryo. In contrast, overexpression of Sox10 in mouse NCCs can maintain its neurogenic potential (Kim et al., 2003; John et al., 2011). Sonic Hedgehog (Shh) is a morphogen with multiple roles in various kinds of organogenesis. It has also been discovered to play an important role in the fate specification of NCC. The results of excision experiments in mice and poultry indicate that the endodermal Shh signal can influence the fate of cartilage differentiation from the inner and outer mesenchymal of pharynx bone (Couly et al., 2002; Aoto et al., 2009), as well as in chick, mouse, and zebrafish development studies (Piotrowski and Nüsslein-Volhard, 2000; Brito et al., 2008). Besides, there are other regulatory signals that have been studied and have been reviewed in more detail (Bhatt et al., 2013).

##### 2.2.3.2 The Formation of Cranial Tendons

One study focused in detail on the development of facial tendons in zebrafish. Zebrafish have two Scleraxis genes, scleraxisa (*scxa*) and scleraxisb (*scxb*). Scxa expression is detected earlier than Scxb during zebrafish development, so Scxa serves as the main Scleraxis gene in zebrafish. The first Scxa transcripts in zebrafish are detectable by 36 hpf (hours post-fertlilization) between the myotomal boundaries along the anterior-posterior axis, which is the period when tendon progenitor cells form. This process requires FGF and TGFβ signaling, similar to the developmental process of limb tendons and axial tendons, as described earlier. In addition, it was discovered that patterning of cranial tendon progenitors requires *cyp26b1*, with the evidence that tenoblasts are disturbed in *cyp26b1* mutant embryos (McGurk et al., 2017). After tendon progenitors are formed, the development of tendons moves on to the next stage at about 60 hpf. At this stage, the tendon progenitor cells gradually differentiate into tenocytes, and then secrete tendon-specific extracellular matrix, the most basic of which is type Ⅰ collagen, which begins to be produced and arranged at this stage to form the inherent form of the tendon. During this process, tenocytes migrate into the specific area consistently until being localized at a suitable position. For example, tendon elements of the mandibulohyoid and hyohyal junctions, and the formation of the sternohyoideus tendons. At 60–80 hpf, the *scxa* and *tnmd* transcripts are coexisted in the cranial area (Chen and Galloway, 2014).

Muscle and cartilage play an important role in the maintainance of Scxa. It was discovered that once the genes involved in cartilage or muscle were knocked out, the expression of Scxa appear abnormal at 72 hpf, indicating that the development of tendons is affected. The aforementioned *cyp26b1* also plays a role in this process, specifically indicating that the loss of *cyp26b1* inhibited the formation of tenocytes condensation and their connection, thereby affecting the tendons’ completeness (McGurk et al., 2017).

Current research on cranial tendon development has focused on zebrafish, and it is uncertain whether the data can be extrapolated to mammals. Additionally, whether cranial tendons are regulated by more specific signaling pathways is unknown and needs further study to verify.

### 2.3 Regulation of Tendon Development

#### 2.3.1 Biological Signals

##### 2.3.1.1 Key Transcription Factors and Relative Specific Proteins

Tendon, ligament and other connective tissues express a famous specific marker Scx, which is highly expressed in tendon progenitors and persists throughout the process of tendon development (Schweitzer et al., 2001). Scx belongs to the proteins widely expressed class I basic helix-loop-helix and is a heterodimer with E12 or E47 proteins by binding to the E-box consensus sequence, serving as a transcription factor during the process of cell differentiation (Cserjesi et al., 1995; Furumatsu et al., 2010). In normal mouse embryos, the presence of Scx allows tendon progenitor cells to condense normally to form tendon primordia, secreting enough extracellular matrix to maintain the correct alignment of tendon cells. In *Scx* ^−/−^ embryos, both the intermuscular as well as force-transmitting type of tendons are affected. Force-transmitting tendons are more serious, whereas the anchor tendon was not affected (Huang et al., 2019). The specific mechanism is mentioned above. In addition, due to the absence of *Scx*, tendon progenitor cells cannot aggregate when they need to condense to form tendon primordia, and remain as loosely arranged mesenchymal cells, thus affecting subsequent tendon development. For example, it was found that in the section of proximal metacarpals in E13.5 mutant mouse models, the distinct condensed layers, divided by ventral progenitors, which will give rise to the flexor digitorium profundus (FDP) and the palmar metacarpal ligament, were not successfully formed and remained confusedly arranged progenitors in mesenchyme as early mesoderm. Besides, the firmest tendon in *Scx-*null mutant mouse, the distal part of the FDP, presents with various extracellular matrix production disorders, including a reduction in tendon collagen quantity and confusion in alignment (Murchison et al., 2007). According to recent research on tendon transcription factors, during the early phase of tenogenic, Scx directly activate 32 target genes, including *Fmod*, *Tnmd*, *Htra3*, *Zfp185*, and *Ssc5d*, and there are 17 genes inhibited by Scx (Liu et al., 2021). Thus, it can be speculated that among all transcription factors involved in tendon development, Scx is a more upstream gene which regulates the functioning of other transcription factors. And it has been shown that Scx is present and important in both the cranial, trunk and limb tendons (Liu et al., 2014). Meanwhile, Scx is also regulated by other signaling pathways. FGFs and TGFβ signaling pathway induce Scx expression, while BMP antagonizes Scx expression in limb tendons and SHH antagonizes Scx expression in axial tendons. The details of the signaling pathway will be described below.

**FIGURE 2:**
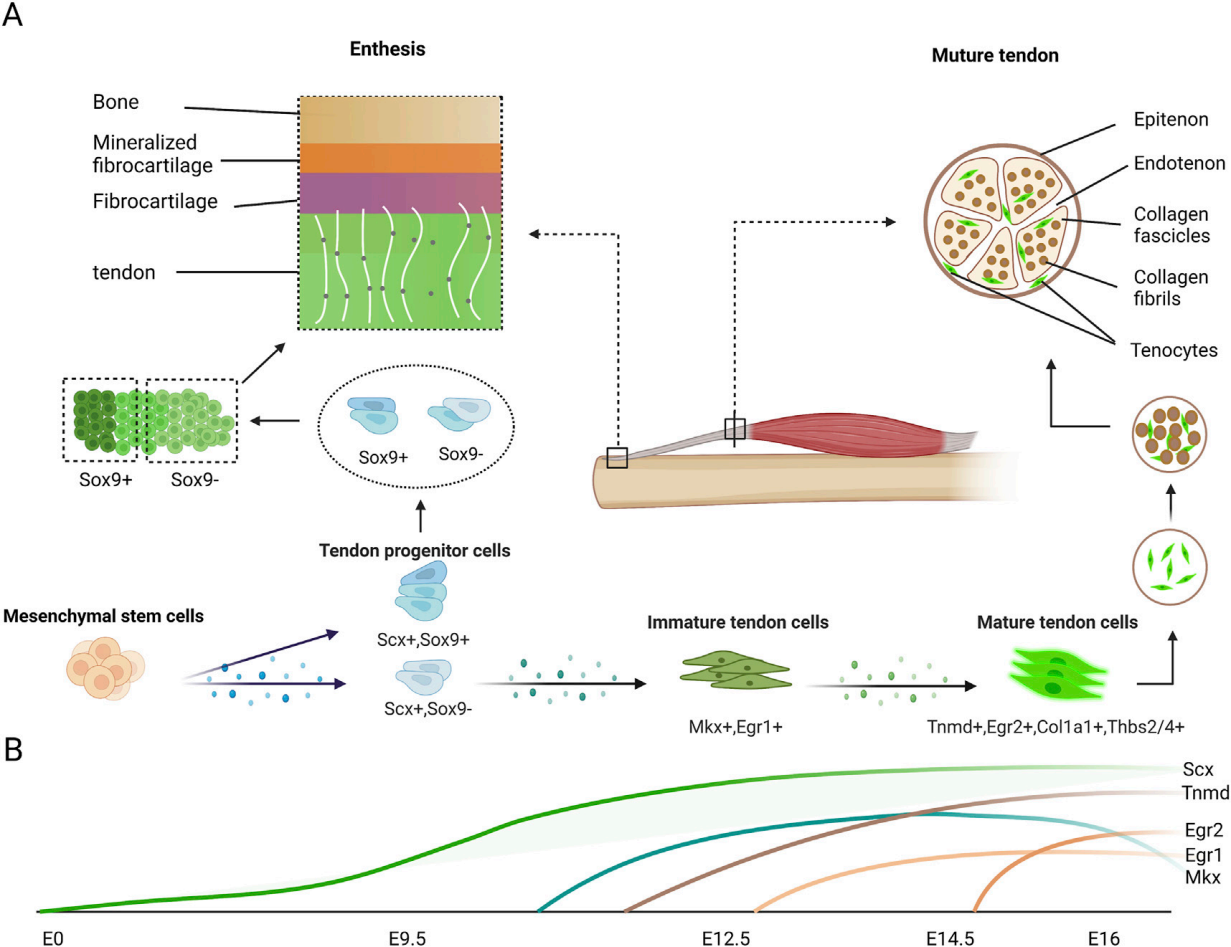
Expression of tendon markers in tenocytes during tendon development. **(A)** Mesenchymal cells differentiate into Scx-expressing tendon progenitor cells, which also partially express Sox9. Scx^+^Sox9^+^ progenitor cells differentiate into the tenocytes which are located near the bone in the enthesis. Other progenitor cells gradually express normal tendon markers during maturation. The classic enthesis is composed of four layers. From tendon to bone end are tendon layer, fibrocartilage layer, mineralized fibrocartilage layer and bone layer respectively. The mature tendon is composed by collagen fascicles which are assembled collagen fibrils, with some tenocytes attached around. **(B)** In mouse limbs, *Scx* expression begin to increase at E9.5 and continue to increase until tenocyte maturation. Slight *Mkx* expression is detectable in tendon at E12.5, after the emergence of *Scx* and robust *Mkx* mRNA expression at E13.5 and E14.5, stages at which the tendon progenitors undergo condensation and differentiation. *Egr1* transcripts are first expressed at E12.5 in Scx domains forming tendon, and they are expressed in long tendons at E16.5. *Egr2* is first detectable in E14.5 limb tendons and is generally expressed in all limb tendons by E16.5. *Tnmd* is highly expressed in E14.5 and is considered a late tendon marker.

Mohawk (Mkx) is a member of the Three Amino acid Loop Extension superclass of atypical homeobox genes. It is also called Iroquois homeobox-like 1 (Irxl), and is expressed in developing tendons (Anderson et al., 2006). A small amount of *Mkx* mRNA expression is observed in the tendon-related region in mouse embryo at E12.5, and *Mkx* mRNA expression become robust after the emergence of Scx at about E13.5 or E14.5, which is the stage that tendon progenitors begin to condensate and differentiate. By E16.5, Scx expression is gradually decreased in the extremities and tail tendons (Liu et al., 2014) (Figure 2). The tail in *Mkx*-null mutant mouse model takes on a wavy shape, and most tendons in *Mkx*-null mutant mouse model are smaller, paler, less vibrant and more hypoplastic than that in wild mouse throughout whole body (Ito et al., 2010; Liu et al., 2010; Kimura et al., 2011). It was also observed smaller collagen fibril size and lower level of type I collagen expression in *Mkx*-null tendons. In addition, down-regulation of molecules such as lumican, decorin, and fibromodulin that attach to the collagen I fibrils and regulate the growth of collagen fibers, was also observed in *Mkx*-null mouse model (Ito et al., 2010; Liu et al., 2010). Therefore, this suggests that *Mkx* is essential for regulating the expression of type I collagen and related matrix in tenocytes. Due to reduction tendon mass and decreased diameter of tendon, the mechanical properties of the Achilles tendon are affected in *Mkx*-null mouse model, thus suggesting functional depression.

Early growth response 1 and Early growth response 2 (Egr1 and Egr2*)* are two transcription factors from the same family, both involved in tendon development process (Lejard et al., 2011; Guerquin et al., 2013). Their sequence is homologous with a gene regulating tendon development in *Drosophila* (Becker et al., 1997). Egr1/2 show distinct expression patterns during tendon development. Egr1 is first expressed at E12.5 in the region of Scx marked and Egr2 is in E14.5 limb tendons. But at E16.5 in the mouse embryo, their expression covered all limb regions (Figure 2). Interestingly, no abnormal tendon phenotype is detected in both *Egr1*-null and *Egr2*-null mouse model (Lejard et al., 2011), demonstrating less prominent effects for Egr compared to Scx and Mkx. However, mutant mouse model of *Egr1* or *Egr2* deficiency appears decrease expression of Col1a1 and reduced number of collagen fibrils within developing tendons, indicating that they participate in tendon differentiation by regulating other transcription factors and collagen-related genes (Lejard et al., 2011). Additionally, it is established that the *Egr* gene has the capacity to promote the expression of tenogenic marker *Scx*, the main collagen in tendon *Col1a1*, and other tendon-related collagens *Col3a1*, *Col5a1*, *Col12a1*, and *Col14a1*. The gene expression research of *Egr1* function inhibition in mouse model has discovered abundant target genes of *Egr1* in connective tissues. An interesting discovery is that the target genes positively associated with *Egr1* are generally related to tendon ECM, while those markers for other tissue differentiation are negatively regulated by *Egr1* (Gaut et al., 2016; Havis and Duprez, 2020). Moreover, it was also demonstrated that the function of *Egr1* to promote tenogenic was consistent in rabbit tendon stem cells (Tao et al., 2015). There is increasing evidence that *Egr1* is also mechanically-sensitive. *Egr1* is activated when mechanical stimulation is applied to 3D-engineered tendons induced in mouse, equine, and human tendons (Yang et al., 2019; Herchenhan et al., 2020).

Transmembrane glycoprotein tenomodulin (Tnmd) is considered as a terminal tendon-related marker and highly expressed at E14.5 in mouse limb tendon cells (Brandau et al., 2001; Shukunami et al., 2001; Jelinsky et al., 2010; Havis et al., 2014). Tnmd is a number of type II transmembrane glycoproteins family. It plays a necessary but not indispensable role in later proliferation and maturation of tenocytes (Figure 2). Although *Tnmd*-null mouse doesn’t appear severe abnormal tendon phenotype, the decreased density and proliferation of tendon cell could be observed *in vivo* (Docheva et al., 2005). Furthermore, it has been discovered that the size of collagen fibril was pathologically increased in *Tnmd*-null Achilles tendons by ultrastructural analyses, taking on the status of impaired collagen fibrilogenesis and premature matrix aging (Docheva et al., 2005). Moreover, self-renewal assays validated that *Tnmd*-null tendon stem/progenitor cells (TSPCs) exhibit significantly decreased proliferative potential *in vitro* (Alberton et al., 2015). And it was found out that cellular senescence was one of the most obvious differences, beginning earlier and manifesting more in *Tnmd*-null model contrasted with wild mouse. Additionally, deficient of *Tnmd* in TSPC results in an abnormal gene expression profile (Alberton et al., 2015). *Tnmd*-null TSPCs appear significant down-regulation of multiple genes, such as collagen-related genes like *Col1a1*, *Col3a1*, and *Col6a1* and the ECM-related genes like *Prg4*, *Thbs4*, and *Comp*. On the contrary, deficient of Tnmd significantly down-regulates of the collagen cross-linking genes like *Dcn*, *Fmod*, *Lum*, and *Lox* (Yin et al., 2019). The above is the study of Tnmd in traditional TSPCs. In recent years, researchers have discovered that Tnmd-positive subpopulation of human adipose tissue-derived stem cells (hASCs) exhibit phenotypical features of tendon progenitor cells and can be biochemically induced towards tenogenic lineage (Gonçalves et al., 2018). Meanwhile, as the source of regeneration cells, Tnmd-positive hASCs is applied in tissue-engineered magnetic cell sheet patches for tendon regeneration (Gonçalves et al., 2017). In summary, Tnmd serves as a downstream transcription factor which regulates tendon collagen and other ECM, and may also have a feedback effect on upstream signals.

Thrombospondins (TSPs, *Thbs*) are deposited in the ECM and are provided with multiple functions to affect vascular, tendon and other types of cells and tissues. A large expression of TSP-4 of the 5 TSP family can be detected in tendons and muscles (Subramanian et al., 2007). A study has experimented the appearance of tendon ECM, particular collagen, and skeletal muscle function and morphology in TSP-4 deficiency mouse (Frolova et al., 2014). In *Thbs4-*null mice, the diameter of collagen fibrils is significantly wider than that in wild mouse, but the organization of collagen is abnormal and the function of tendons is affected (Frolova et al., 2014). Besides, it has been found that TSP-4 is indispensable for muscle connection and ECM organization in vertebrates. TSP-4b deficiency in zebrafish model causes muscle detachment during the process of contraction because of the deficiency in laminin gathering and inhibition of Itg signaling (Subramanian and Schilling, 2014). Thus, TSP-4 is necessary for the regular fibril organization and the interaction with other ECMs. It also contributes to the maintenance of correct ECM composition in tendon, and functions as an ECM scaffold, serving as an important factor in the late stage of tendon development.

In conclusion, as the markers of tendon tissue, these genes are specifically expressed in different stages of tendon development and function as regulatory factors in the process of tendon development. (Figure 2). In the meantime, our understanding of these genes is still in its infancy, and much remains to be discovered.

##### 2.3.1.2 Signaling Pathway

*TGF-β Signaling*. Bioinformatics analysis of the transcriptome of tenocytes show TGFβ signaling pathway is one of the most activated pathways in tenocytes during limb tendon development in mice (Havis et al., 2014). Despite the activation of TGFβ signaling induces Scx and other transcription factors expression (Pryce et al., 2009), the early effect of TGFβ signaling in tenogenic differentiation of mouse is not determined completely, because *in vivo* studies have shown that at E11.5, the mutant embryos (*Tgfb2* ^*−/−*^ *;Tgfb3* ^*−/−*^ embryos) also display normal tendon phenotypes (Pryce et al., 2009). And, Scx was already expressed normally in tendon progenitor cells at this time, indicating that the early stage of tendon differentiation had been successfully induced. This indicated that TGFβ induction of tendons did not occur at an early stage or there are other ligands involved in this process. In subsequent tendon development, TGFβ plays an essential role. It can promote further differentiation of tendon progenitor and increased tendon specific genes expression in the cells via canonical TGFβ intracellular signaling pathway, SMAD2/3, and end up with mature tenocyte (Havis et al., 2014). The expression of Scx also decreases in zebrafish embryos by blocking canonical TGF-β intracellular signaling pathway (Chen and Galloway, 2014). And whether TGFβ or Smad2/3 signaling pathway are blocked in explant mouse limb models, tendon-related genes including *Scx*, *Col1a1*, and *Col1a2*, are observed down-regulated (Havis et al., 2014). *Smad3*-null mice showed a tendon orientation defective phenotype (Berthet et al., 2013); however, the defective phenotype of the *Tgfb2/Tgfb3* double knockout mutant mouse model is more severe than that of *Smad3* knockout mice, suggesting that Smad3 is not the only downstream molecule of TGFβ signaling pathway involved in tenogenic process (Murchison et al., 2007). In addition, TGFβ also contributes to maintain the fate of tendon cells. Recently, it has been found that when TGFβ signal is inactivated after mouse tendons mature, the differentiated tenocytes will lose tendon-specific markers and dedifferentiate into primitive tendon stem/progenitor cells (Kaji et al., 2020).

Even though all of the above studies have demonstrated positive effects of TGFβ on tendon development, there are still some studies showing TGFβ also promotes chondrogenesis, which is the opposite to the tenogenic process (Roark and Greer, 1994; Gañan et al., 1996). Research has shown that TGF-interacting factor Tgif1 and SKI-like oncogene SnoN as potential candidates for modulating this process. Tgif1 has been identified to involved down-regulation of Sox9 and Agn and up-regulation of Scx, and Tnmd through the Smad pathway, thus leading to tenogenesis (Lorda-Diez et al., 2009). Beyond that, more explanations for the phenomenon are still being investigated.

Other than the classical TGFβ pathway, GDF-5, -6, -7 and-8 (also known as myostatin), which are ligands of TGFβ superfamily, also contributes to proper tendon formation. In *GDF*-5, -6 and -7 deficient animal models, the collagen structure and biomechanical capacity have been altered in tendons. Whereas, tendon cells with additional GDF5 activation showed greater capacity of collagen secretion (Dines et al., 2011), while MSC in the same state promoted the expression of tenogenic genes and inhibited the expression of chondrogenic genes (Tan et al., 2012; Qu et al., 2018). In *Gdf-7* deficient mice, it is observed normal tendon phenotype and upregulated expression of GDF-5, indicating that GDF-5 levels may replace the role of GDF-7 to maintain the regular development of tendon in the situation of GDF-7 deficient. In another study, GDF-7 promotes the mesenchymal stem cells to differentiate towards tendon progenitors of spindle shaped, and these cells express increased level of Scx an Col1a1 *via* TGFβ signaling pathway (Wang et al., 2005; Mikic et al., 2008). Myostatin is known to be expressed normally in muscle cells during embryo development process, and functions to negatively regulate muscle production (McPherron et al., 1997). But in tendons, myostatin activation in mouse limb tendon-derived fibroblasts can induce the expression of tenogenic genes like Scx and Tnmd (Edom-Vovard et al., 2002; Mendias et al., 2008), demonstrating its opposite effect on tendons as it does in muscles.

*FGF Signaling*. FGF signaling has also been demonstrated to contribute to early Scx expression in tendon progenitors, but its effects on chicken embryos and mouse embryos appear to be opposite (Havis et al., 2016). In chicken embryos, FGF plays a positive role in Scx expression (Brent et al., 2003; Brent et al., 2005). FGF is proved to activate ERK MAPK intracellular pathway to take effect on tendon progenitors in syndetome of somitic, of which the effector Pea3 and the modulator Sprouty2 are both discovered in areas that tendon progenitors distributed (Smith et al., 2005). On the contrary, in mouse models, the function of FGF signaling pathway is weakened during the development stage of tenocytes, and inhibition of ERK MAPK signaling pathway could induce the expression of Scx, Col1a1, and Col1a2 in mouse limb tendon progenitors (Havis et al., 2014). In humans, as in mammals, the role of FGF signaling in tendon development is still not completely understood, but tends to be consistent with that in mice.

*Wnt Signaling*. It has been proven that tendon formation is disrupted after ectodermal removal (Schweitzer et al., 2001; Edom-Vovard and Duprez, 2004). And it is widely believed WNT signaling pathway contribute to his ectodermal influence on connective tissue differentiation. However, WNT/ß-catenin signal is present both in the ectoderm and mesoderm mesenchyme, and investigations via the *Wls* gene, which regulates the secretion and distribution of various Wnts ligands, reveals that the function of ectodermal Wnts is to keep the multipotency ability of distal mesenchyme progenitors. By contrast, the elimination of mesoderm mesenchymal Wls intends to prevent distal mesenchyme from differentiating (Zhu et al., 2012). In other words, Wnts signals from ectodermal and mesoderm mesenchymal play an opposite role in mesenchymal progenitors. The function of WNT/ß-catenin signaling is to coordinate the cell fate of connective tissue formation and maintain a pool of progenitors with sub-ectodermal mesenchymal cells. The mesenchymal cells progress to tendon cell differentiation program when the levels of the WNT/ß-catenin signaling are reduced. It is indispensable and sufficient to suppress WNT/ß-catenin signaling for the induction of tenogenic gene Scx (ten Berge et al., 2008; Garcia-Lee et al., 2021). This indicates that the WNT signaling pathway may be the key to induction of the tendon differentiation process. There are other studies that showed Wnt/β-catenin signaling suppress the expression of Scx in tendon-derived stem cells *via* eliminating the TGFβ-associated regulation of Scx expression by antagonize the activation of TGF-β, as well as down-regulate the expression of *Mkx* and *Tnmd* (Kishimoto et al., 2017). Moreover, Wnt3a, one of the ligands of Wnt signaling pathway, is proved to upregulate the expression of Six2 in autopod developing tendons of chick embryos (Yamamoto-Shiraishi and Kuroiwa, 2013). In conclusion, the role of Wnt signaling extends throughout the process of tendon development and there is still a lot to discover.

*mTOR Signaling*. mTOR (mechanistic target of rapamycin) is a threonine/serine protein kinase, and is a part of mTOR complex 1, which serving as a bridge to connect metabolic processes and nutrient signals that are essential for cell development and widely regulating biological activities including cell proliferation, differentiation, and metabolism (Ben-Sahra and Manning, 2017). During tendon development, the persistent activation of mTORC1 in tenocytes is vital for the ECM production and maintain. In various studies, it was found that mouse models that loss its mTORC1 function specifically in tenocytes showed reduced tendon thickness and down-regulated expression of Mkx, Tnmd, SLRPs, Dcn, Col1a1, and Fmod. On the other hand, mouse model in which the function of mTORC1 was specifically enhanced exhibited positive tendon phenotype including angiogenesis in tendons and hyperproliferation of cells (Lim et al., 2017; Cong et al., 2018). Another study demonstrated that mTOR contributed to the synthesis and remodeling of collagen in tendons under mechanical stimulation (Mousavizadeh et al., 2020). Moreover, it can also block non-tenocyte differentiation which is caused by over-activated mechanical stimulation in some pathologic conditions to prevent tendons from tendinopathy. *In vitro* it was demonstrated that mechanical loading activated mTOR signal in rat patellar TSPCs in a stretching manner, and consequently, the cell proliferation and non-tenogenic differentiation of TSPCs was decreased significantly, as indicated by the low expression of chondrogenic makers and osteogenesis markers (Nie et al., 2021).

*Retinoic Acid Signaling*. Retinoic acid (RA) signaling was researched early in chick embryos, and it was discovered that tenocytes in chick embryo contained cellular retinoic acid binding protein (CRABP) and that retinoids modulated collagen synthesis in tendon and osseous tissue (Oikarinen et al., 1986). A later study observed that retinoic acid receptor agonists could be selected as effective inducers of nuclear Scx in small molecular sieves, indicating that RA signaling participates in the specification of tendon cells (Webb et al., 2016). Recent research explains this in more detail. It was demonstrated that RA initially contribute to the regulation of tendon progenitors, with mESCs undergoing neural differentiation when RA signaling is activated, while differentiating into tendon progenitors of paraxial mesoderm when RA signaling is inhibited (Kaji et al., 2021). RA further regulates tendon progenitor cells, differentiating into the tenogenic lineage when RA signaling is activated, with reverse differentiation into fibrocartilage (Kaji et al., 2021). Besides, it was found that the variations of retinoids concentration in endogenous local region promoted the formation of tendon condensations and attachment sites within the extraocular area (Comai et al., 2020). However, RA inhibition experiments leads to defects in muscle belly segmentation and myotendinous junction formation of avian limb (Rodriguez-Guzman et al., 2007).

#### 2.3.2 Mechanical Stimulation

The complex mechanical/physical environment that embryonic tendon cells experience will affect their later tissues stiffness and dynamic load. These physical signals also positively contribute to the tenogenic process in embryo. Initially, studies demonstrated a phenotype of reduction in tendon size and degeneration of tendon matrix in a motionless condition of paralysis chick embryo, as well as decreased expression of tenascin-C (Germiller et al., 1998; Mikic et al., 2000). With the deficiency of skeletal muscle, early tenogenic induction and progenitor differentiation are not affected in chicks, but the subsequent process of tendon development will not take place (Kieny and Chevallier, 1979; Kardon, 1998). These results indicate that the presence of muscles may serve as the inducer of mechanical stimulation, or secretor of soluble factors, or both. Subsequently, some studies *in vitro* and *in vivo* have shown that developed tendons can exhibit better phenotypes when subjected to certain mechanical stimuli, including up-regulated tenogenic genes (e.g., scleraxis, Tnmd, tenascin-C, collagen types I and III) (Kuo and Tuan, 2008; Xu et al., 2011; Xu et al., 2012; Zhang and Wang, 2013) and increased matrix production or increased collagen fibril size and physical properties (Kalson et al., 2011). These stimuli can be quasi-static variable, coded loading, or shear strains. Notably, Scx is mainly upregulated by tension rather than compression force among various kinds of mechanical stimulations (Takimoto et al., 2015).

This phenomenon of tendon sensitivity to mechanical stimulation is also closely related to TGF-β/Smad2/3 and FGF/ERK MAPK signaling pathways, which are currently considered to be downstream signals of mechanical stimulation. It has been shown that mechanical stimulations activate the TGF-β/SMAD2/3 pathway to regulate Scx expression *in vitro* (Maeda et al., 2011). While FGF4 functions to counteract the decreased Scx in chick limbs that lack mechanical stimulation during development. Recent research has further supported this view, in immobilised chick limbs, FGF4 activates Scx expression, while TGFβ2 maintains Scx, Tnmd and Thbs2 expression (Havis et al., 2016). Egr1 may be an intermediate-mediated factor. It has been established that Egr1 is able to be activated by shear stress, and subsequent studies have proven the effects of Egr1 on tendon development as mentioned above. More direct evidence has been discovered in some 3D-engineered tendons models that Egr1 as the downstream of mechanical signals to control tendon gene expression (Yang et al., 2019; Herchenhan et al., 2020). Because the down-regulation of Egr1 is concomitant with the down-regulation of Tgfb2 genes (Gaut et al., 2016) and Egr1 is directly bound to Tgfb2 promoter regions (Guerquin et al., 2013), researchers came up with the opinion that Egr1 can activate Tgfb2 as a downstream signal of physical mechanical stimulations, and in turn regulate tenogenic genes later (Havis and Duprez, 2020).

Mechanosensing and mechanotransduction mechanisms in embryonic tenocytes emerges through direct cell-to-cell connections (Banes et al., 1999; Richardson et al., 2007) (e.g., cadherins and gap junctions) or cell-to- ECM adhesion molecules (Schwartz, 2010) (e.g., integrins). Cell cytoskeletal components in the downstream (e.g., nonmuscle myosin II (NMMII) heavy chain proteins and actin) may also take effect in the mechanical environment (Clark et al., 2007).

**FIGURE 3:**
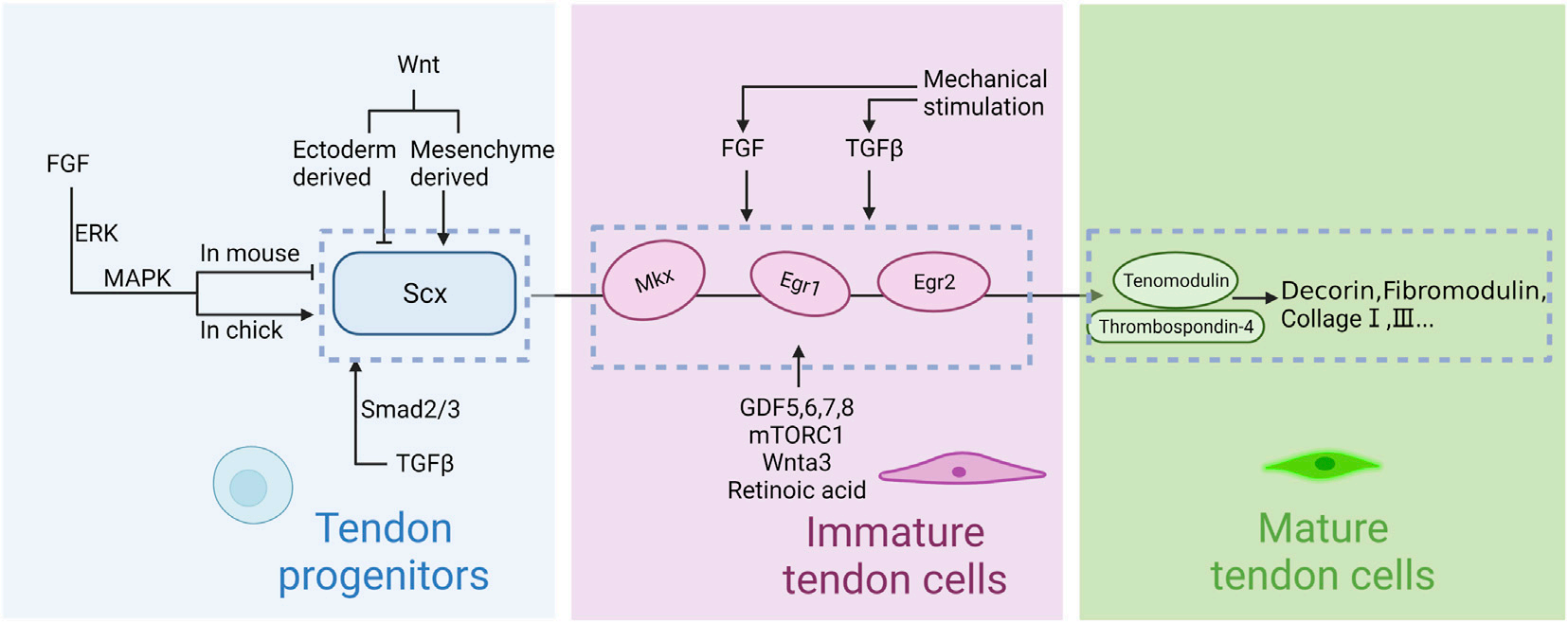
Regulation of tendon development in embryogenesis. Scleraxis (Scx) is the first signaling molecule implicated in tendon progenitor cell initiation, whereas Mohawk (Mkx) and early growth response 1 and 2 (Egr1, Erg2) are secondary signals for tendon differentiation and maturation. Thrombospondins (Thbs) and tenomodulin (Tnmd) are upstream factors which regulate collagen and matrix. FGF, TGFβ and Wnt signaling pathways regulate the induction of Scx. GDF5,6,7,8, mTORC1 and Wnta3 regulate the upstream transcription factors. Mechanical stimulation is involved in regulation via the TGFβ and FGF signaling pathway. Notably, the effects of FGF on chicken embryo and mouse embryo appear to be opposite. Mesenchymal progenitors are maintained by ectodermal Wnts and repressed by mesenchymal Wnts.

In summary, several known signaling pathways and transcription factors work together to regulate various stages of tendon development. Even though the relationships and functions of these transcription factors are unclear and they cannot be connected into a complete pathway at present, the upstream and downstream relationships among some factors are relatively clear (Figure 3). Research in this area is still ongoing.

## 3 The Formation and Development of Tendon-Bone Interface

The multilayered structure of the enthesis originate at the ends of the tendon and bone during the tenogenic process, composed of tendon, fibrocartilage, calcified fibrocartilage and bone. Under the regulation of TGFβ signaling, the fibrocartilage primordium forms after tendon tissue and before the bone tissue and a contiguous area of progenitors expressing both Scx and Sox9 (Scx^+^/Sox9^+^) are induced with an unknown mechanism (Blitz et al., 2013). As embryo develops, these progenitors which are closed to the tendon differentiate into Scx^+^ tenocytes, whereas those closed to the end of bone eliminate the Scx expression and become only Sox9 positive chondrocytes, and the middle region consists of cells that express Scx or express Sox9 in a regular order (Akiyama et al., 2005; Soeda et al., 2010; Sugimoto et al., 2013). During this process, Bmp4, which derived from Scx^+^ cells, promotes the progenitors to differentiate towards chondrocytes (Blitz et al., 2009). Those chondrocytes originated from Scx^+^/Sox9^+^ progenitor partly go through endochondral ossification process to form the bone tissue layer of mature enthesis. In terminal embryo, Gli1 positive cells are present at the immature tendon-bone interface to produce connective matrix and structure, thus promoting the development of enthesis (Dyment et al., 2015; Schwartz et al., 2015; Felsenthal et al., 2018). In the area of craniofacial complex, it was also demonstrated that the Scx^+/^Sox9^+^ progenitors and consequently differentiated cells function together to the forming of tendon-bone interface sites and was associated with FGF signal. Elimination of Fgfr2 expression in NCCs-derived progenitors of mandible changes induction of Scx^+^/Sox9^+^ progenitors and intercept Notch-Dll1 signaling to induce their mistakenly differentiation into chondrocytes (Roberts et al., 2019) (Figure 2).

## 4 Tendon Development Guidance for Tendon Repair

Up to now, the clinical therapy for tendon injuries generally includes surgical treatment and conservative treatment, the former contains sutures, autologous transplantation, artificial transplantation, tendon to bone fixation and the latter contains physiotherapy, drug injection systemic treatment. Despite remarkable progress achieved in operative and rehabilitation treatments, functional recovery remain limited in dealing with the problems of gap formation, adhesions, and rupture (Shen et al., 2018), and there is a lack in the intrinsic regenerative ability to completely recover to the state before injury (Walia and Huang, 2019). Scientists and clinicians are working to make tendon repair more optimal and achieve complete regeneration.

### 4.1 Tendon Repair Process

As previous research studies have demonstrated, the process of tendon repair is different in adults and embryos. When a tendon is damaged at the embryonic stage, it can regenerate in the same way that tendons develop. However, the scar-associated healing process occurs in adults (Voleti et al., 2012).

**FIGURE 4:**
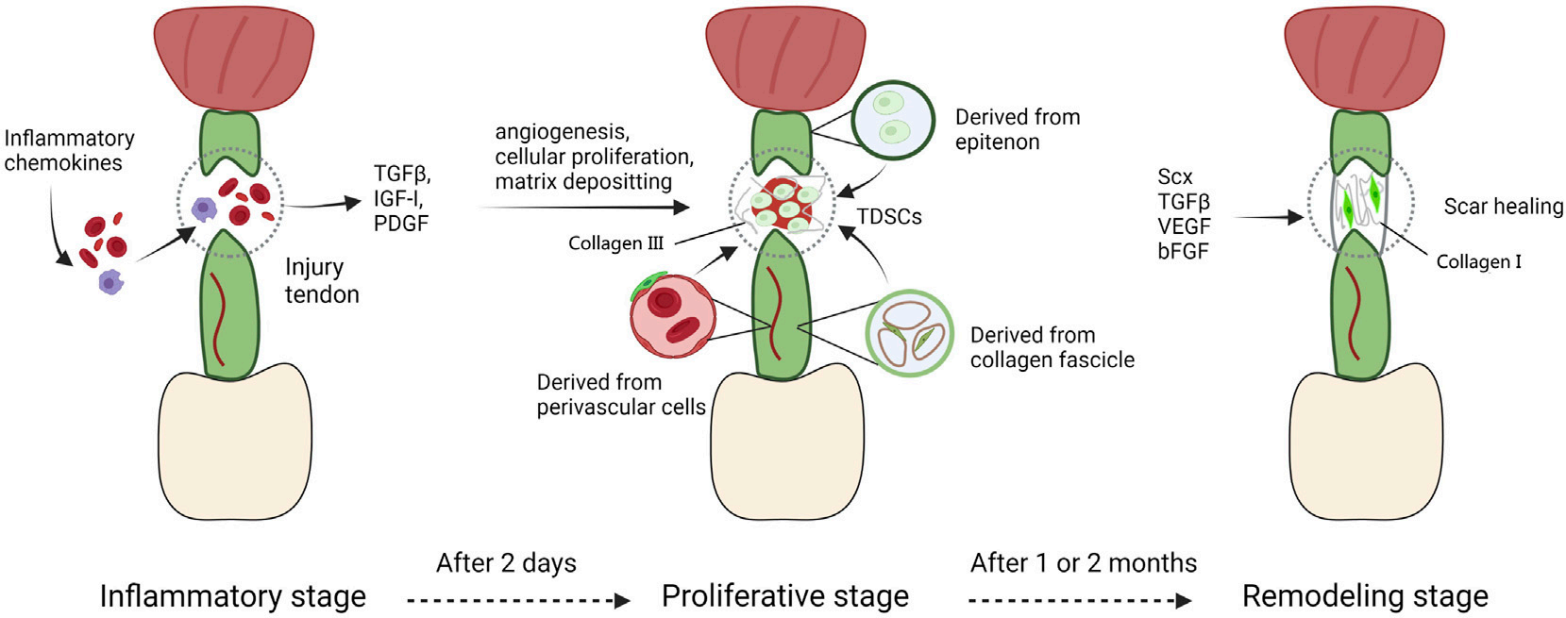
The tendon repair process. The response to adult tendon injury is composed of three blurred-defined stages. The first stage, defined as inflammatory stage, spans a couple of days typically. The injured area is infiltrated with erythrocyte, leukocytes, and platelets secreting key growth factors and endothelial chemoattractants. During the next stage, macrophages synthesize and release growth factors, and induce cells to recruit towards injury region, and change their role from phagocytosis to reparation with a couple of days. In the meantime, tenocytes secrete a serious of matrixes consist mainly of collagen III. At this time, bFGF and VEGF expressed from recruited cells like tenocytes, fibroblasts and inflammatory cells reaches a peak, thus promoting cellular proliferation and angiogenesis. In the final stage, main activity that happens is a process of remodeling. A great amount of collagen I begins to be synthesized, and the ECMs deposited among the injured area become more organized. However, the cell density and synthetic activity in repaired region are not able to recover to normal conditions. This stage appears roughly one or two months after the injury and persistent for more than 1 year.

The response to adult tendon injury is composed of three blurred-defined stages (Hope and Saxby, 2007). The first stage, defined as inflammatory stage, spans a couple of days typically. The injured area is infiltrated with erythrocyte, leukocytes, and platelets secreting key growth factors and endothelial chemoattractants (TGFβ, IGF-I, and PDGF) (Molloy et al., 2003). Fibrin clots are assembled to guarantee transitional stiffness, at the same time macrophages phagocytose necrotic segments, TSPCs migrate to the injured region and are induced to proliferate, particularly in the endotenon and epitenon (Gelberman et al., 1991; Molloy et al., 2003). During the next stage, also appeared as the repair or proliferative stage and starting about 2 days after injury, the character of repair process is abundant of synthesis activity directed by tenocytes as well as macrophages. Macrophages synthesize and release growth factors, and induce cells to recruit towards injury region, and change their role from phagocytosis to reparation with a couple of days (Leadbetter, 1992). In the meantime, tenocytes secrete a serious of matrixes consist mainly of collagen III. But it’s temporary and mechanically inferior than normal matrix in tendons. At this time, bFGF expressed from recruited cells like tenocytes, fibroblasts and inflammatory cells reaches a peak, thus promoting cellular proliferation and angiogenesis (Chang et al., 1998; Molloy et al., 2003; Bedi et al., 2012). VEGF is highly expressed as well, synergistically inducing angiogenesis to offer nutrients, recruited cells, and additional cell factors to the injured region (Bidder et al., 2000). In the final stage, main activity that happens is a process of remodeling. A great amount of collagen I begins to be synthesized, and the ECMs deposited among the injured area become more organized. However, the cell density and synthetic activity in repaired region are not able to recover to normal conditions. This stage appears roughly one or 2 months after the injury and persistent for more than 1 year. The repaired tendon shows scarring phenotype and can never entirely recover to the natural biomechanical properties before injury (Leadbetter, 1992) (Figure 4).

### 4.2 Methods of Improving Tendon Healing

With current established treatment modalities, the natural repair of tendons is only manifested as imperfect scar repair. Therefore, researchers have tried to improve tendon repair for decades. Theoretically there are two ways in which we can alter the imperfect postnatal healing of tendon. One is to change the entire process of tendon repair from scratch, so that it is not scar-associated repair as it is known today in adults, but rather tendon regeneration at the embryonic stage. This approach is arguably the most perfect, fundamental solution to the problem, but it is also the most challenging, and has so far only been achieved in animal models through complex genetic manipulation (Paredes et al., 2018). Hence, this approach is a long way from being applied clinically. Therefore, while carrying out research on the first approach, the second approach to intervene in the repair process is also the focus of current research. Therapeutic approaches to improve tendon healing include biomechanics stimulation, growth factors, biologics and stem cells (tissue engineering) (Leong et al., 2020). Biomechanics stimulation involves a battery of conservative intervene that can be used to promote tendon repair. These methods include ultrasound, cryotherapy, physical therapy, and magnetic fields (Xu et al., 2014; Lu et al., 2016). Many researchers attempted to enhance tendon healing through various growth factors that contribute to tendon healing and development (Liu et al., 2011). The most popular and typical biologic is platelet-rich plasma (PRP), an autologous blood product that is obtained by drawing peripheral venous blood containing concentrated platelets and plasma but removing erythrocyte (Filardo et al., 2018). MSCs used for restoration of tissues *in vivo* had been put forward and carried out since the early 1990s. Similar methods to promote tendon repair had been researched since 1993. It is also the one with the best prospects at the moment (Fu et al., 2019). These four methods are not exclusive to each other, and a growing number of studies showed that combining two or more of these can result in better effects.

### 4.3 Regulation of Tendon Repair

#### 4.3.1 The Role of Tendon Stem Progenitor Cells in Tendon Healing

The discovery of TSPCs in tendon tissues that possess tenogenic differentiation potential and regenerative abilities brings about brand-new possibility for tendon repair.

The initial study to investigate TSPCs suggest that their niche locate among the tendon collagen fascicles and ECM such as fibromodulin and biglycan offer a proper environment for them, which is distinct from other identified stem cell niches, such as perivascular niche for neural stem cells, bulge niche for skin stem cells, and osteoblast niche for hematopoietic stem cells. Besides conventional markers associated with typical stem cells, TSPCs express tenogenic markers such as Scx, and express tendon-related markers Col1a1 and Tnmd, demonstrating a newly identified subpopulation of resident tendon cells (Bi et al., 2007). Later, studies of impaired or overloaded tendon models revealed that the epitenon serves as another origination of TSPCs (Dyment et al., 2013; Dyment et al., 2014; Howell et al., 2017). During mouse development, epitenon cells are observed to surround tendons after distinct tendons are formed and express the marker *Tppp3* (Staverosky et al., 2009). After birth, the epitenon can be distinguished from normally differentiated tenocytes by detection of laminin, platelet derived growth factor receptor α (PDGFRα), and α-smooth-muscle-actin (αSMA), which are only expressed in former (Dyment et al., 2013). Meanwhile, it has been observed that progenitor cells of Prg4 lineage and αSMA lineage are recruited to participate the process of tendon healing, and that paratenon cells, which is not Scx-lineage cell under homeostasis, also act to the damage by expressing tenogenic genes Scx and depositing ECM to fill up the defect area (Dyment et al., 2013; Dyment et al., 2014). TSPCs derived from both areas of tendon form tendon-related structures *in vitro* and show pluripotency, indicating that tendon tissue contains two distinct origination of stem cells (Mienaltowski et al., 2013), corresponding to TSPCs subpopulations derived from tendon fascicle and epitenon. Latest researches indicated that perivascular or pericytes cells derived from the surrounding vascular system might be the third origination of TSPCs. And they might be the direct origination of TSPCs derived from epitenon. TSPCs derived from paratenon express high levels of pericyte and vascular markers like *Musashi1* (*Msi1*), *Cd133*, and *Endomucin* (*Emcn*) than that in TSPCs derived from tendon fascicles (Mienaltowski et al., 2013). It has been confirmed that Scx, Msi1, αSMA, Cd133, and Nes is present in perivascular TSPCs by immunostaining of the perivascular TSPCs isolated from human tendon microvessels (Tempfer et al., 2009). A former study discovered some assertive evidence that TSPCs can also be explanted from adjoining tissues (Huang et al., 2021). Besides, a study based on the zebrafish models revealed that totally ablating tendons could be well-restored by progenitors derived from neighboring tissues. Thus, there exists a group of progenitors serves to coordinate tendon regeneration at the surrounding cartilage or muscle adhering area (Niu et al., 2020). This study corroborates evidence that surrounding tissues might contain the respective progenitor or stem cell subpopulations of adjacent tissues. In summary, these results indicates that there might be three or more different subpopulations of TSPCs.

One study showed that TSPC accounts for about 4% of the total number of tenocytes (Bi et al., 2007). Normally, TSPCs from both the epitenon and tendon fascicles are involved in the healing of tendon, corresponding to the extrinsic and intrinsic response to tendon injury respectively (Bi et al., 2007). Intrinsic recruitment of Scx-lineage cells are crucial for regeneration of tendon, but it only occurs in embryonic and early postnatal tendons (Howell et al., 2017). While in adult tendons, Scx-lineage cells undergo abnormal differentiation towards the chondrogenic lineage rather than directed recruited to injured area (Howell et al., 2017). During the process of adult tendon repair, TSPCs from epitenon play a major role. In response for injury, Tppp3^+^Pdgfra^+^ cells will be recruited to the damaged area, in which they differentiate into tenocytes and lose their pluripotency, to repair and fill up it (Harvey et al., 2019). Also, fibrosis in the process of tendon healing might occur due to this subpopulation (Harvey et al., 2019). Inflammation reaction is an important section of the tendon healing and may ultimately affect repair outcomes. The effects of HIF-2α, Prostaglandin E2 and IL-1β on TSPCs, promote enhanced expression of tenogenic genes (Huang et al., 2021). After inflammatory stimulation, both embryonic and postnatal tendon cells exhibit similar tenogenic commitment, but the latter also expressed up-regulated catabolic enzymes and inflammatory mediators (Li et al., 2019). In summary, TSPCs serve as the vital cells to migrate to the area of injury, proliferate and then express tendon-related, pericyte-related, and pluripotent markers to regulate the process of tendon healing and remodeling (Huang et al., 2021). TSPCs from the tendon proper participate in healing of embryonic and early postnatal tendons, contributing to the complete regeneration of embryonic and early postnatal tendons. While TSPCs from epitenon contribute to fibrosis during healing of adult tendons.

#### 4.3.2 Signaling Pathways

##### 4.3.2.1 Factors Implicated in Both Development and Repair

In addition to playing a key role in tendon development and tenocyte differentiation, TGFβ is also a known inducer of fibrotic scar formation various tissues, containing adult tendons (Katzel et al., 2011; Thomopoulos et al., 2015). It is well established that TGFβ drive the myofibroblast differentiation and exceeding TGFβ ligand released after injury can also induce the apoptosis of tenocyte (Maeda et al., 2011). Given these conflicting effects of TGFβ signaling in tendon development and scarring, the direct role of TGFβ signaling during tendon regeneration is remain unknown. There has been a lot of research trying to resolve this ambiguity (Kaji et al., 2020). For example, Kaji et al. (2020) studied TGFβ-dependent and TGFβ-independent processes involved in neonatal tendon regeneration respectively. It was found that early proliferation of Scx^+^-lineage tenocytes and activation of aSMA^+^ cells do not depend on TGFβ signaling. However, proliferation of Scx^−^-lineage cells, and subsequent tenogenic cells recruitment (composed of both Scx^+^-lineage and Scx^−^-lineage cell sources), as well as functional recovery depended on TGFβ signaling (Kaji et al., 2020). Additionally, it was also indicated that the application of TGF-b3 to tendon cells up-regulated the expression of Smad7and down-regulated the expression of Smad3, consequently minimized extrinsic scarring and decreased tendon adhesion to improve tendon repair (Jiang et al., 2016), which was consistent with another study that showed inhibition of Smad3 improved the healing process in a rotator cuff injury model (Wang et al., 2021).

FGF is also the signal that participates in both tendon development and repair. It has been reported that FGF-2 and platelet-derived growth factors (PDGF-BB) combined application tocanine flexor tendon fibroblasts can significantly promote the process of collagen production and cell proliferation and (Thomopoulos et al., 2005). Low concentration of FGF-2 induces a continuous response process of human BMSCs, during which cell proliferation was significantly activated in the early phase and cell differentiation was stimulated in the late phase (Hankemeier et al., 2005). The differentiation towards tendon lineage cells of MSCs is also promoted by the delivery of FGF-2 through various scaffolds (Sahoo et al., 2010; Ker et al., 2011). During the healing of rat rotator cuff, the models treated by FGF-2 demonstrated remarkably heightened histological appearance and biomechanical strength. In addition, FGF-2 contribute to healing of enthesis via promote tendon stem/progenitor cells proliferation, thus bringing about a better recovery of the healing rotator cuff (Tokunaga et al., 2015).

In summary, these signals contribute to both tendon development and repair, although the specific effect is not completely the same. These differences may be caused by differences in ligand, environment, cell type and other factors, which need further research.

##### 4.3.2.2 Factors Involved Only in Repair

Besides the above two growth factor signaling pathways, vascular endothelial growth factor (VEGF), insulin-like growth factor-1 (IGF1), connective tissue growth factor (CTGF), and platelet-derived growth factor (PDGF) are also crucial factors in tendon repair. In several conditions, these growth factors also regulate the expression of tendon-related gene *in vitro*, although these effect for tendon biology are not completely proved *in vivo*. The application of CTGF can recruit a TSPC subpopulation with CD146 positive and stimulate both proliferation and tenogenic differentiation (Lee et al., 2015). IGF-1 serves to induce ECM production and recruit fibroblasts to the wound region, while PDGF strengthens translation and transcription processes, thereby up-regulating expression of other involved growth factors. High expression of VEGF stimulates angiogenesis to provide nutrients, extrinsic cells, and additional growth factors to the injury region (Molloy et al., 2003; Leong et al., 2020). In summary, these factors mainly mediate the initial inflammatory response and cell chemokines of the early stage of injury, and work synergistically to initiate the healing process.

#### 4.3.3 Transcription Factors and Relative Specific Proteins

As one of the most important transcription factors in tendon development, Scx also contribute to tendon repair and regeneration. Expression of Scx in progenitors is initially induced by TGF-β signaling. In the case of normal injury, progenitor cells from the paratenon or other region migrate to the site of injury in order to respond to repair. However, in *Scx*-deficient mouse, it was indicated that progenitors migrated to the wound area but failed to bridge the defect because of the destroyed ECM assembly. Mechanistically, *Scx*-deficient progenitors show much more chondrogenic potential with up-regulation of *Sox9* coactivator PPAR-γ coactivator-1α (PGC-1α) than normal progenitors, and analysis based on knock-in models reveals that the Sox9 is remarkably suppressed by forced expression of full-length Scx. Accordingly, *Scx*-deficient mouse models form cartilage-like tissues that develop ectopic ossification in wound area (Sakabe et al., 2018). In another study, Scx shows crucial effects in adult tendon response for mechanical loading. It is demonstrated that after supraphysiological overload of the plantaris tendons, the cross-sectional area of neotendons in *Scx*-null mice was smaller than that in control mice. And it was also demonstrated that the ability of CD146^+^ pericytes to differentiate into tenocytes was reduced in *Scx*-null mice (Gumucio et al., 2020). In addition, there are studies that have reported mesenchymal stem cells genetically modified with Scx could express more tendon-related genes and less osteo- and adipose-related genes (Alberton et al., 2012), which improved regeneration in an animal model of rotator cuff injury (Gulotta et al., 2011).

It had been indicated that S100 calcium-binding protein A4 (S100a4) marks a subset of tendon cells and is a inducer of tendon scarring. Besides, adult Scx^−^lineage cells reside in the area that will become scar tissue and are assembled into a cellular bridge during tendon healing. In addition, it has been demonstrated that S100a4 and Scx are expressed by different populations of tendon tissue during the condition of healing and homeostasis, with Scx being expressed in the organized bridging tissue but S100a4 located throughout the whole scar area (Best and Loiselle, 2019). Mechanistically, S100a4 drives fibrotic tendon healing primarily through a cellular dependent process, with S100a4 haploinsufficiency decreasing myofibroblast and macrophage numbers at the site of injury, thus promoting regenerative tendon healing (Ackerman et al., 2019a).

The role of Egr1 in tendon regeneration was first investigated in a stem cell study (C3H10T1/2 stem cells) in which forced Egr1 expression resulted in the commitment of MSCs to tendon differentiation *via* activated expression of Scx, Col1a1, Col1a2 and other collagens and molecules that make up ECMs of tendon tissue. As Scx, the activation of Egr1 suppresses the differentiation of MSCs towards the osteogenic or adipogenic lineages (Guerquin et al., 2013).

Biglycan (Bgn) has been identified as one of the critical constituent parts of the TSPCs niche and might have relation to tendon development. At a proper concentration, Bgn promotes the expression of the tendon-related markers Thbs-4 and Tnmd through both the transcription and translation process. At the meantime, it inhibits the expression of osteogenic and chondrogenic markers Acn, Runx2, and Sox9. These are achieved through the BMP7/Smad1/5/8 pathway pathways (Zhang et al., 2019).

Tnmd is crucial for prevention of fibrovascular scar formation and adipocyte accumulation during early stage of tendon repair. A study showed that scar tissue in *Tnmd*-null tendon contained fibronectin, cartilage oligomeric matrix protein (Comp), and augmented matrix sedimentation of biglycan, with altered profile of macrophage and reduced amount of CD146-positive cells. Additionally, *Tnmd*-null TSPCs exhibits excessive adipogenic differentiation accompanied with significantly increased transcription level of peroxisome proliferator-activated receptor gamma (Pparγ) and lipoprotein lipase (Lpl) (Lin et al., 2017).

In summary, many factors involved in tendon development are increasingly being indicated to participate in tendon repair and regeneration, although they take effect at different stages.

## 5 Conclusion

To date, research studies on tendons have been more and more comprehensive and rigorous. We have a general idea of how tendons develop in animal models. The relationships and differences between tendon repair and development have also been recognized. We are always convinced that the understanding of tendon development will provide us a basis for the development of more effective and scientific treatments of tendon injury. Thus, more specific aspects of tendon development still require exploration. From the earliest discovery of *SCX* as a marker gene of connective tissue such as tendon, to the discovery of syndetome, the source of trunk tendon, we still cannot plot a complete signaling network, even though we have elucidated a variety of signaling pathways and factors that regulate the development of tendon tissue. Because the upstream and downstream relationships of some transcription factors have not yet been identified, it is uncertain whether there are still signaling molecules involved in tenogenesis that have not yet discovered. For example, *EGR1* is regarded as the downstream gene of *SCX*. However, when the effect of *Egr1* is inhibited, the expression of *Scx* is also affected. The current explanation is that *Egr1* affects *Scx* expression by regulating the ligand of TGFβ, but other possibilities may exist. In addition, certain signals exert opposite effects in different animal models. For example, as mentioned earlier, the FGF signaling pathway play distinct roles in limb tendon development within the mouse and chick models. More research studies are needed to carried out to understand the role of the FGF/ERK pathway in tendon development.

Understanding the process of tendon repair and regeneration is just as important as understanding the process of tendon development. At present, the problem is that the repair of adult tendons can only achieve scar-associated repair with limited functional recovery, but not optimal repair like that of embryonic tendons. Although some animal models have exhibited self-regeneration of adult tendon, the process and technology are complex and challenging. Therefore, the focus of current research is still how to intervene during the repair process of tendon to achieve the possibility of tendon regeneration. Through the comparison of tendon development and repair mentioned above, we can find that there are many similarities and some obvious differences between these two processes. Thus, at a macro level these are really two similar physiological processes, but the difference in the key factors leads to the difference in their outcomes. On the one hand, it is vital to discover these regulatory factors, because only in this way can the fundamental regulation guide the development of tendon repair to a more perfect direction. Researchers have made some progress in this area. On the other hand, how to efficiently operate these regulatory factors is also a problem that needs to be faced in the future. Ethic, cost and practicability must be considered when applying these results to humans. With advances in stem cell and tissue engineering technology, coupled with studies of regulatory signals from tendon development, this goal is gradually being achieved (Ekwueme et al., 2016; Wu et al., 2016; Veronesi et al., 2017).
